# Microsaccade rate as a measure of drug response

**DOI:** 10.16910/jemr.12.6.12

**Published:** 2019-10-03

**Authors:** Elliot Hampsey, Paul G. Overton, Tom Stafford

**Affiliations:** Department of Psychology, University of Sheffield, UK

**Keywords:** Eye movement, eye tracking, microsaccades, Attention Deficit Hyperactivity Disorder, ADHD, caffeine, superior colliculus, sensitivity analysis

## Abstract

In 22 human subjects we measured microsaccade count across 60 brief fixation trials both pre- and post- administration of 300mg of caffeine. There was a statistically significant reduction in average microsaccade count post-caffeine administration, with a moderate effect size (Cohen’s d of 0.42). Microsaccade count was stable within individuals across time points (Pearson’s r of 0.89). Sensitivity analysis suggests that the pre/post caffeine effect size is robust to choice of parameters used to identify microsaccades. Bootstrap resampling suggests that both the pre/post-caffeine difference and the across-time stability within individuals could be reliably assessed with far fewer trials. The results support the use of microsaccade count as both a trait measure of individual differences and a state measure of caffeine response. We discuss the results in the context of the theory that the superior colliculus is central to the generation of microsaccades and hence that microsaccade rate may be a useful assay for at least some drug-induced changes at the level of the colliculus: a potentially useful tool in the development of therapies for disorders that may involve collicular dysfunction such as ADHD.

## Introduction

Microsaccades are miniature fixational eye movements approximately 6 to 25-milliseconds in duration, through 1-120 arcminutes (up to 2 full degrees of rotation), that occur at a rate of about 1-2 per second. Electrophysiological studies have demonstrated the role of the rostral pole of the superior colliculus in microsaccades in primate models (1,2). For example, collicular neurons have been shown to be active for microsaccades in monkeys (3), and Goffart & colleagues observed a significant drop in the microsaccade rate during fixation after deactivating the rostral deep SC of monkeys (4). Experimental inactivation of certain neurons in the monkey colliculus reduces the rate of microsaccades, but only if those neurons were implicated in movements of ≤5 degrees of arc (1). In humans, Attention Deficit Hyperactivity Disorder (ADHD) - a disorder that has been argued to involve a dysfunction at the level of the superior colliculus (5)- is associated with alterations in the production of microsaccades (although this evidence is not entirely consistent (6, 7, 8, 9, 10). 

Evidence suggests that collicular activity is modulated by a range of neurotransmitter systems, one of the least explored – at least in humans – is the adenosine system. Intraperitoneal administration of an adenosine deaminase inhibitor has been shown to enhance neural activity in the superficial grey layer of the superior colliculus following electrical activation of the optic tract in the anaesthetised rat in vivo (11), as has the direct application of adenosine to the superficial grey (12). The superior colliculus expresses both A1-type (13) and A2-type adenosine receptor (14), and the excitatory action of adenosine itself can be reproduced by the direct application of selective A1-type and A2-type adenosine receptor agonists (14). In humans, adenosine action in the colliculus has been poorly researched, however, in the context of microsaccades, pilot work by Collins and colleagues (15) suggests that caffeine ingestion results in fewer microsaccades, as might be expected following the removal of an excitatory influence in the colliculus. Caffeine acts at all four distinct adenosine receptors thus far identified, both in the rat and human brain, although affinity data suggest that it’s actions are likely to be mainly mediated via and interaction with A1 and A2 receptors (16), both of which are expressed in the colliculus (13, 14, 17).

The present study follows on closely from the earlier pilot work by Collins and colleagues (17) by exploring the effects of caffeine consumption on the production of microsaccades, this time in a full study, as an indirect assay of the potential excitatory action of adenosine in the human superior colliculus. We recorded microsaccade rate during a simple sustained fixation task in a population of healthy adults, both before and after the consumption of caffeine. The primary hypothesis was that there would be a decrease in microsaccade rate when measured after caffeine consumption compared to before. As well testing the effects of caffeine on microsaccade production in a full study, we also had a more general aim of examining the utility of microsaccades as an assay of collicular function. Here, our intention was to scrutinize the stability of the measure in the absence and in the presence of a pharmacological manipulation. Demonstrating that microsaccades are stable over time pre-drug and stable over time (although their parameters may change) post-drug, would underscore their usefulness as an index of human collicular function and its modification.

## Methods

### Participants

Twenty-seven participants (13 male) were recruited via intra-university advertising at the University of Sheffield. The final group of 22 participants (taking into account exclusions) had a mean age of 29.4 years ranging from 19-47 (SD = 8.57). Three participants were excluded from the final analysis as calibration problems prevented binocular tracking. Data for these participants were collected monocularly, but ultimately excluded from final analyses in order to standardise the data set. Two further participants were excluded due to strabismus that prevented successful calibration, leading to a total of five exclusions. All subjects were right-handed, free from diagnosed neurological disorder or injury, and free from stimulant class medications (including methylphenidate). Although none of the participants had a current diagnosis of ADHD, their relatively low levels of ADHD-like traits were confirmed by their low scores (mean 31.05; standard error 2.09) on the Adult ADHD Self-Report Scale (ASRS; 18).

Participants were asked not to ingest caffeine in the two hours before the study, both upon registration and in an email reminder of their study appointment the day before. They were told specifically to avoid caffeinated drinks such as coffee and energy drinks, as well as caffeine tablets. All participants confirmed upon arrival, via the signing of the consent form, that they had not consumed any of the above examples of caffeinated beverages/tablets.

Participants received £10 for taking part in the study. The subjects all gave their informed consent to take part in the experiment and the procedures were conducted in accordance with both the University of Sheffield’s ethics committee guidelines, and the Declaration of Helsinki. 

### Eye tracking

The eye tracking was performed using an Eyelink 1000 (SR Research Osgood, ON, Canada), which recorded data binocularly at 500Hz. The fixation task was created from scratch using the experiment builder (SR Research Osgood, ON, Canada) software packaged with the Eyelink hardware. The experiment was displayed to the participants on a 19" DMW LCD monitor with screen dimensions of 1024x768 and a 60hz refresh rate. The monitor was placed 65cm away from the participant headrest. The experiment took place inside an enclosed room with the curtains drawn and light turned on in order to achieve a constant illumination level. 

### Procedure

A 9-point grid was used for calibration. Validation was deemed successful if the disparities from calibration were less than 1 degree. If validation was unsuccessful, calibration was repeated. The process took approximately 2 minutes for each participant. Drift correction using a central fixation point was performed before every trial, to counter small inaccuracies in calibration produced by pupil dilation and participant movements. Calibration was performed twice for each participant; at the beginning of each block. 

The task was administered after calibration and validation were complete. Following the initial drift correction, a fixation cross appeared in the centre of the screen, which disappeared once the participant fixated upon it for 300ms. The removal of the fixation cross triggered the appearance of an orange target cross at one of 6 target positions with horizontal displacements of, respectively, -8.6, -4.3, 0, 0, +4.3, +8.6 degrees, and vertical displacements of 0, 0, +6.4,-6.4,0,0 degrees. Each target position was used 5 times in each block, with the order randomised to prevent predictive saccades. The participants were tasked with fixating upon the target cross for 5.5 s until it disappeared. Research has suggested that fixations of 10 s or longer can to cause pupil contractions, which are significant confounding factors with regards to pupil centration and thus eye tracking calibration (19). With that in mind, a presentation time of 5.5 s was considered to be a good compromise between the need to acquire sufficient data and avoiding the disruptive effects of pupil contractions. The removal of the target cross was followed by 2.25 s of blank screen until the next trial began with a new drift correction calibration point. 

Each eye tracking session consisted of 2 blocks of 30 trials. The removal of the black fixation cross relied upon the participant fixating upon it successfully, which was of course reliant on both participant compliance and eye tracker accuracy. In the rare instances this was not achieved punctually, the eye tracker was recalibrated. The eye tracking sessions took an average of approximately 12 minutes each, with a range of 10-15 minutes. 

Once the first eye tracking session was complete, the participants were given tablets containing 300mg of caffeine to ingest with water. Caffeine was given in the form of one 200mg tablet (myvitamins.co.uk) and two 50mg tablets (Pro-plus, Lane Health Products Ltd, Gloucester, UK) (for 300mg total) in which the only listed active ingredient was caffeine. This dosage was chosen as 300mg of caffeine has been shown to significantly increase dopaminergic activity in the putamen and ventral striatum (20), which means such a dose would likely serve our intended purpose of stimulating oculomotor circuitry. Further, moderate doses of caffeine (~300 mg, 4mg/kg) have been shown to have behavioural effects in various cognitive domains, including attention, reaction times, and executive function (see [21] for review). 

 Participants had a 45-minute break between blocks, as caffeine bioavailability has been demonstrated to peak at 99% after approximately 45 minutes (22). Upon the completion of this break period, the participants repeated the eye tracking session in exactly the same way as before.

### Data analysis

Raw eye tracking data was preprocessed in Python using PyGaze (23). Microsaccade identification was performed using the Microsaccade Toolbox 0.9 (24). Unless otherwise specified the parameters used for identifying microsaccades were a minimum duration of 6 samples (12 ms) and a velocity threshold scaling factor of 5. All raw data and scripts to reproduce the analysis are available at https://osf.io/je8hf/

## Results

### Main Results

The mean count of microsaccades pre-caffeine administration was 1.40 (sd 0.52), compared to a post-administration mean of 1.18 (sd 0.43). This was a statistically significant reduction (t(21) = 4.367, p = 0.00027) with a standardised effect size of 0.42 (Cohen’s d, a small to medium effect according to [25]).

**Figure 1.**
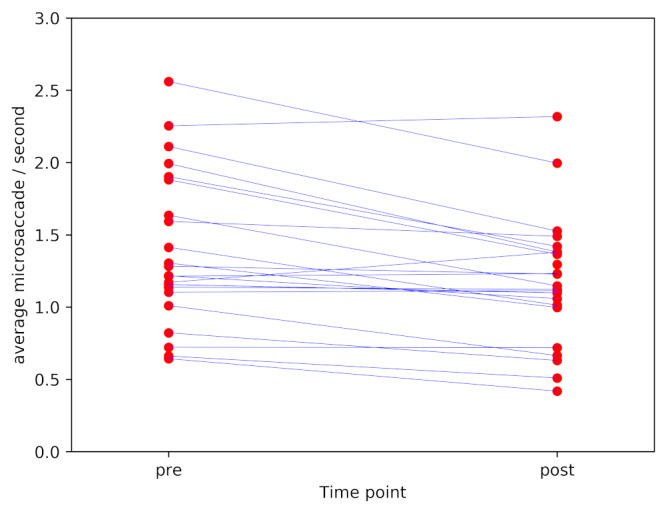
Mean microsaccade count/second pre and post caffeine administration (lines connect measurements from the same individual pre and post).

The pre-post correlation was 0.89 (Pearson’s r, p<0.0001). The stability of microsaccade count as an individual difference measure is visualised in Figure 1.

There was no significant correlation between the ASRS score of ADHD traits and either pre-administration microsaccade count or reduction in microsaccade count (r = 0.18, -0.13 respectively, p = 0.43, 0.57). We note that 15 of our 22 participants had ASRS scores in the narrow range 20-40.

### Sensitivity Analysis

**Figure 2.**
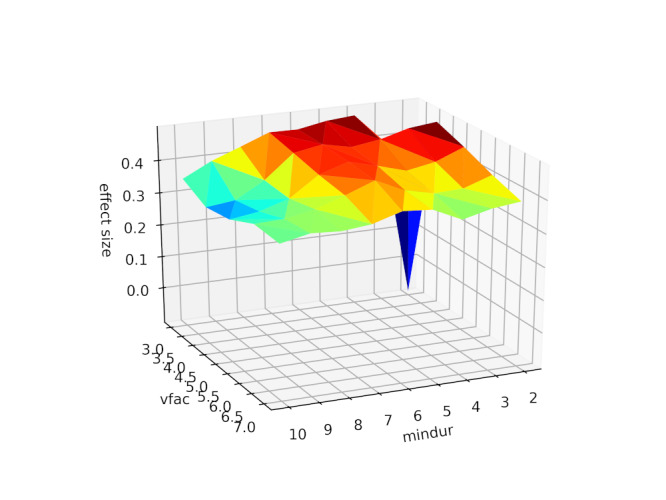
Surface plot of effect size (z-axis) against microsaccade identification parameters (minimum duration, ‘mindur’, x-axis, and velocity threshold scaling factor, ‘vfac’, y-axis). For full description of these parameters see (24).

The prior analysis used standardised parameters for microsaccade identification (see method). We confirm that the apparent sensitivity of microsaccade count to caffeine administration is not due to the specific parameters picked to identify microsaccades by recalculating the pre and post caffeine microsaccade counts from the raw data with values of the minimum duration parameter between 2 and 11 samples (6 was our default) and velocity threshold scaling factor between 3 and 8 (5 was our default). The results are shown in Figure 2, confirming that the pre-post difference is not an artifact of choice of microsaccade identification parameters.

Note that effect size is well above zero for all combinations of identification parameters, except for the most conservative.

**Figure 3.**
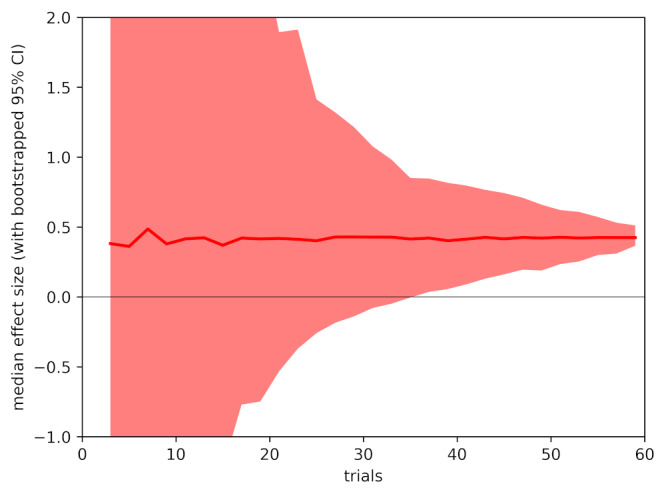
Sensitivity analysis, showing mean (line) and bootstrapped 95% confidence range (shaded area) for the effect size of pre-post caffeine microsaccade count difference for analysis based on 3 – 60 trials.

**Figure 4.**
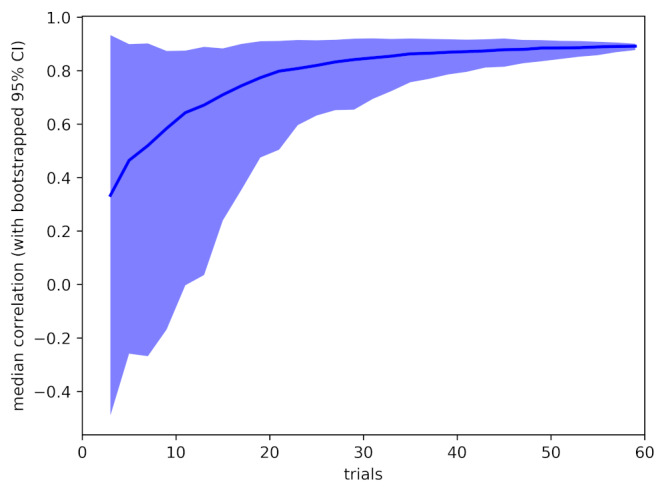
Sensitivity analysis, showing mean (line) and bootstrapped 95% confidence range (shaded area) for the pre-post caffeine microsaccade count correlation for analysis based on 3 – 60 trials.

It is also possible to use the collected data to ask how stable the obtained results would be if less data had been collected from participants. Our standard analysis uses data from all 60 trials, but we are interested in how stable the results would have been if we had collected fewer trials. Using bootstrap resampling, we calculate 95% confidence ranges for the two summary statistics – pre-post correlation of microsaccade count within subjects, and effect size of pre-post microsaccade count difference. The results are shown in Figure 3 and Figure 4.

**Figure 5.**
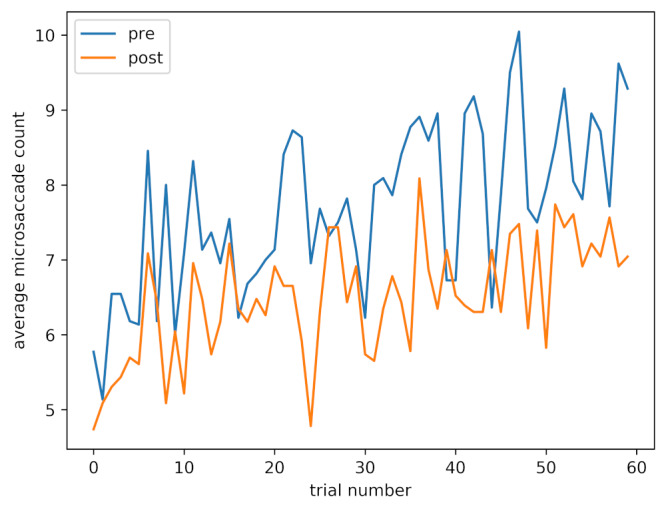
Average microsaccade count against trial number, pre and post caffeine administration.

Figure 5 shows average microsaccade count for each trial, both pre and post caffeine administration. Although later trials, both pre and post, are associated with a higher microsaccade count, the main effect of a lower count post-caffeine is also clearly visible.

## Discussion

As predicted, caffeine administration reduced microsaccade count, on average. Additional analysis of the results suggests that microsaccade count has promise as an individual difference measure. There was a high consistency of microsaccade count across our testing sessions, as is desirable for a trait measure. The mean difference pre/post caffeine administration suggests that microsaccade count is sensitive to caffeine, as predicted by earlier work (15) and by analysis of the circuitry involved in saccade generation (5). We have shown that this difference is robust to choice of parameters used to identify saccades. This suggests that microsaccade count may also be suitable as a state measure. 

The sensitivity analysis suggests that both the individual stability of microsaccade count and the sensitivity of caffeine (as reflected by the between session difference) could be reliably detected with approximately half the number of trials we used.

Microsaccades can be measured with low participant effort and are suitable as a repeated measure (i.e. have negligible learning or fatigue effects). 

Our interest in microsaccades originated because we hoped they would provide a good index of collicular function. This is not directly tested by the present work, which is focused on the sensitivity and stability microsaccade count as a measure. Nonetheless, the present results support the potential of microsaccade count for further investigation as a possible biomarker of drug response, possibly at the collicular level. If microsaccade rate reflects collicular activity levels the measure may provide a means of initially screening potential pharmacotherapies for disorders with a collicular component. Attention Deficit Hyperactivity Disorder may be one such condition, and in that regard it is interesting that adults presenting with ADHD report regular and increased consumption of caffeine through a variety of beverages over time (26), and adolescents with a diagnosis of ADHD are nearly as twice likely to consume caffeine than are adolescents without ADHD (27).

Circumstantial support for the idea that ADHD may involve collicular hyperactivity include that those higher in ADHD-like traits demonstrate heightened sensitivity to peripheral cues (28), have an altered temporal integration window for multisensory stimuli (29), and report higher levels of sensory sensitivity and processing problems (30).

Limitations of the present research which can be addressed in future work include assessing the stability of microsaccade count over longer time periods, and directly comparing caffeine administration with placebo. Factors such as fatigue, attention and arousal have a known influence on microsaccade rate (31), and so could plausibly be involved in producing the observed pre- and post- caffeine administration differences. Ultimately any strong causal inference about the effect of caffeine on microsaccade rate must wait for direct confirmation with an experiment with a non-drug control condition. It would also be worthwhile to investigate if microsaccade count can be reliably assessed during active viewing (i.e. without using fixation trials, which can be monotonous for participants).

## Ethics and Conflict of Interest

The author(s) declare(s) that the contents of the article are in agreement with the ethics described in http://biblio.unibe.ch/portale/elibrary/BOP/jemr/ethics.html and that there is no conflict of interest regarding the publication of this paper. 

## Acknowledgements

We wish to thank all participants in the experiment, Maria Panagiotidi, Edwin Dalmaijer for discussion and advice on analysis, and Joel Martin at SR Research for eyetracking support. 
